# Zinc-alpha-2-glycoprotein Secreted by Triple-Negative Breast Cancer Promotes Peritumoral Fibrosis

**DOI:** 10.1158/2767-9764.CRC-24-0218

**Published:** 2024-07-05

**Authors:** Surbhi Verma, Stephanie D. Giagnocavo, Meghan C. Curtin, Menusha Arumugam, Sandra M. Osburn-Staker, Guoying Wang, Aaron Atkinson, David A. Nix, David H. Lum, James E. Cox, Keren I. Hilgendorf

**Affiliations:** 1 Department of Biochemistry, University of Utah School of Medicine, Salt Lake City, Utah.; 2 Metabolomics, Proteomics and Mass Spectrometry Core, School of Medicine, University of Utah, Salt Lake City, Utah.; 3 Huntsman Cancer Institute, University of Utah, Salt Lake City, Utah.

## Abstract

Obesity is a modifiable predisposition factor for postmenopausal breast cancer. This suggests a localized, reciprocal interaction between breast cancer cells and the surrounding mammary white adipose tissue. To investigate how breast cancer cells alter the composition and function of adipose tissue, we screened the secretomes of 10 human breast cancer cell lines for the ability to modulate the differentiation of adipocyte stem and progenitor cells. The screen identified an adipogenic modulator, zinc-alpha-2-glycoprotein (ZAG/AZGP1) that is secreted by triple-negative breast cancer (TNBC) cells. TNBC-secreted ZAG inhibits adipogenesis and instead induces the expression of fibrotic genes. Accordingly, depletion of ZAG in TNBC cells attenuates fibrosis in white adipose tissue and inhibits tumor growth. Further, high expression of ZAG is linked to poor prognosis in patients with TNBC but not in patients with other clinical subtypes of breast cancer. Our findings suggest a role of TNBC-secreted ZAG in promoting the transdifferentiation of adipocyte stem and progenitor cells into cancer-associated fibroblasts to support tumorigenesis.

**Significance:**

Functional screening of breast cancer secretomes revealed that triple-negative breast cancer promotes fibrosis in the adipose tissue microenvironment by secreting zinc-alpha-2-glycoprotein and promoting the transdifferentiation of adipocyte stem cells into myofibroblasts.

## Introduction

Approximately one in eight women will develop breast cancer in their lifetime, and more than 40,000 women in the United States die of breast cancer every year (1). Breast cancer is clinically subdivided into subtypes based on the expression of three cellular markers: estrogen receptor (ER), progesterone receptor (PR), and human epidermal growth factor receptor 2. Triple-negative breast cancer (TNBC) does not express any of these markers, accounts for 10% to 20% of all breast cancers in the United States, and is associated with poor prognosis (2).

Several factors can increase the risk of developing breast cancer, including modifiable risk factors such as obesity (3). The role of obesity in breast cancer has received significant attention in recent years, in part owing to the increased prevalence of obesity in general, with half of adults globally and 70% of adults in the United States being considered overweight or with obesity (4). Obesity significantly increases the risk of postmenopausal breast cancer, with each 5 unit increase in BMI linked to a 12% increase in risk (5). In addition, obesity increases the risk of large, high-grade tumors, metastasis, and recurrence regardless of menopausal status (6, 7). Obesity is a risk factor for both estrogen receptor-positive and TNBC, suggesting that a multitude of mechanisms underlie the obesity–breast cancer link (8).

Obesity is characterized by an expansion of white adipose tissue, the major tumor microenvironment of breast cancer. Thus, there is significant interest in elucidating how the cellular constituents of adipose tissue accelerate breast cancer cell growth. White adipose tissue is comprised of energy-storing endocrine cells called adipocytes, adipose stem and progenitor cells (ASPC), endothelial cells, and immune cells (9). The role of adipocytes in promoting breast cancer has received considerable attention in recent years. Cancer-associated adipocytes release adipokines, chemokines, growth factors, and extracellular matrix proteins that contribute toward tumor development and metastasis (10, 11). Several studies have highlighted the importance of the ratio of two adipokines, leptin, and adiponectin, to the progression of postmenopausal breast cancer as well as TNBC (12). Adiponectin, whose expression decreases with obesity, inhibits breast cancer growth (13). In contrast, leptin, whose expression is increased with obesity, promotes breast cancer growth (14). Similarly, resistin secretion, which is linked to obesity and diabetes, can promote breast cancer (15). Finally, Maguire and colleagues (16) showed that obese adipocytes synthesize and secrete increased amounts of creatine that promotes the growth of the TNBC cell line E0771 in obese mice.

Other cellular components of adipose tissue may similarly promote breast cancer growth, although the underlying mechanisms have not been described in detail. Breast cancer promotes increased circulation and recruitment of ASPCs to the tumor microenvironment, particularly in obese patients (17). Notably, orthotopic xenografts of breast cancer cells in combination with ASPCs show increased tumor growth and metastasis, further enhanced if ASPCs were isolated from obese individuals (18, 19). The depletion of ASPCs in the microenvironment inhibits tumorigenesis (20). How ASPCs promote breast cancer growth remains unclear. Possible mechanisms include differentiation into additional cancer-associated adipocytes, secretion of angiogenic or inflammatory factors, or transdifferentiation into fibroblast-like cells to increase tumor microenvironment stiffness (21). Not all of these mechanisms are mutually exclusive and their relative contributions to breast cancer growth may depend on clinical subtype and tumor grade. We do not yet know how breast cancer regulates the fate and function of ASPCs.

Fibrosis describes the stiffening of tissue due to the deposition of extracellular matrix (ECM) components such as collagen (22). Studies have shown that breast cancer cells are more proliferative and invasive when grown *ex vivo* in stiff ECM and *in vivo* when injected into fibrotic tissue (23, 24). Accordingly, increased fibrosis in the tumor microenvironment is linked to increased breast cancer aggressiveness. Cancer-associated fibroblasts (CAFs) are thought to be the main culprit of fibrosis in the tumor microenvironment. However, recent single-cell sequencing has shown that there are multiple subtypes of CAFs, potentially reflecting different origins (25). This includes mature adipocytes, which were shown capable of becoming more fibroblast-like in the presence of breast cancer cells *ex vivo* (26). ASPCs are another source of CAFs, and recent single-cell transcriptomic analyses have identified ASPC subpopulations with fibroblast gene expression signatures (27, 28). Notably, ASPCs isolated from obese adipose tissue or from adipose tissue adjacent to breast cancer show increased fibroblast-like gene expression, likely contributing to the increased amounts of adipose tissue fibrosis observed in both settings (28–31). The mechanisms underlying this pathogenic remodeling and how breast cancer cells may instruct the fate of ASPCs in the surrounding adipose tissue toward fibroblasts remain unclear.

In this study, we screened the secretomes of 10 human breast cancer cell lines, encompassing all clinical subtypes, for their ability to modulate adipogenic differentiation of ASPCs. The secretomes of all the TNBC cell lines tested potently inhibited adipocyte differentiation. Using mass spectrometry, we identified that a subset of TNBC cells secreted zinc-alpha-2-glycoprotein, ZAG/AZGP1, an adipokine previously linked to adipocyte lipolysis and cancer cachexia (32). We showed that ZAG treatment of ASPCs promotes the expression of fibrotic genes. Accordingly, loss of ZAG dramatically reduces fibrosis in the tumor microenvironment and impedes tumor growth.

## Materials and Methods

### Breast cancer cell–conditioned media

Breast cancer cells were seeded at ∼ 30% confluency in DMEM + 10% Fetal Calf Serum (Thermo, 10438026) + 1% Pen-Strep in 5% CO_2_ at 37°C. At 90% confluency, spent media was aspirated off; cells were gently washed with PBS and incubated with DMEM only (no serum) for 24 to 72 hours to be conditioned with cell-secreted factors. No cell death was apparent by imaging. At the end of incubation, conditioned media containing the breast cancer cell secretome was collected and passed through a 0.44 micron filter to remove any possible cell debris. Collected conditioned media was immediately flash frozen in liquid nitrogen, and stored in −80°C.

For heat inactivation experiments, conditioned media was incubated at >95°C for 10 minutes and then allowed to cool at room temperature. Heat-inactivated conditioned was then mixed in a 1:1 ratio with DMEM + 20% FBS + differentiation cocktail for *in vitro* adipogenesis experiments (see below).

Amicon ultra 100 K centrifugal spin columns were used for size fractionation. Briefly, conditioned media was transferred to a 100 kD Amicon spin column and centrifuged at 4,000 *g* for 20 minutes. Filtrate and supernatant were collected separately and flash frozen in liquid nitrogen. For the *in vitro* adipogenesis experiments, the supernatant (top fraction) was reconstituted with DMEM to the initial volume of conditioned media loaded on the spin column. Reconstituted supernatant and filtrate were separately mixed in a 1:1 ratio with DMEM + 20% FBS + differentiation cocktail for *in vitro* adipogenesis experiments (see below).

### Isolation of primary ASPCs

Mice were euthanized with isoflurane and secondary cervical dislocation. Inguinal white adipose tissue was dissected out and lymph nodes were removed. Tissue was removed and minced in HBBS (Gibco, 14025-92) and incubated in Collagenase Buffer [3,000 U/mL type II collagenase powder (Sigma, C6885), 100 U/mL DNase 1 (Roche, 10104159001), 1 mg/mL poloxamer 188 (P-188; Sigma P5556), 1 mg/mL BSA, 20 mmol/L HEPES buffer, and 1 mmol/L CaCl_2_ in Medium 199 with Earle’s salts (Sigma, M4530)] for 15 minutes shaking (250 rpm) at 37°C. Equal volume of FBS was added to collagenase buffer to neutralize. Digested samples were strained through a sieve followed by centrifugation at 4°C (1,300 rpm for 10 minutes).

The cell pellet supernatant was resuspended in FACS buffer (PBS, 2% FBS, 1% PenStrep, 1% GlutaMAX) and strained through a 70 micron filter, followed by centrifugation at 4°C (1,300 rpm for 8 minutes). Pellet was resuspended in 37°C Red Lysing Buffer (Sigma, R7767) for 1 minute, followed by inactivation using DMEM complete growth media (DMEM, 10% FBS, 1% PenStrep, 1% GlutaMAX). Cells were centrifuged at 4°C (1,300 rpm for 7 minutes) and resuspended into 5 mL of wash solution [300 μg/mL DNase in sterilized water, 70% DPBS with MgCl_2_ and CaCl_2_ (Sigma, D8662)], followed by centrifugation at 4°C (1,300 rpm for 6 minutes). Cell pellet containing primary ASPCs were washed with FACS buffer and resuspended into 200 µL FACS Buffer.

For flow sorting ASPCs, antibodies were added to 200 µL of cell suspension in FACS buffer and incubated for 30 minutes on ice. Cells were then centrifuged at 4°C (1,300 rpm for 5 minutes) and resuspended in 500 µL FACS buffer. Cells were then sorted on BD FACS Aria Cell sorter. ASPCs (Lin- CD34^+^ Sca1+ CD29^+^) were resuspended in DMEM containing 10% FBS, 1% Pen/Strep, and 1% GlutaMAX. Antibodies used were the following: CD45 PE-Cy7 (eBioscience, 25-0451-82), CD31 PE-Cy7 (eBioscience, 25-0311-82), Ter119 PE-Cy7 (eBioscience, 25-5921-82), CD34 PE (BD Pharmingen, 551387), Sca1 APC (Biolegend 108112), and CD29 FITC (Invitrogen, 11-0291-82).

### *In vitro* adipogenesis

3T3-L1 cells were grown to confluency in DMEM containing 10% bovine calf serum (Sigma, 12133C), followed by another 2 days at confluency in DMEM containing 10% bovine calf serum. Adipogenesis was then induced using DMEM containing 10% FBS and differentiation cocktail consisting of 1 μg/mL insulin (Sigma, I5500), 1 µmol/L Dexamethasone (Sigma, D4902), and 0.5 mmol/L 3-isobutyl-1-methylxanthine (IBMX; Sigma, I7018) (differentiation media). After 2 days of differentiation cocktail, media was changed to DMEM containing 10% FBS and 1 μg/mL insulin (maintenance media). Maintenance media was changed every 2 to 3 days for a total differentiation time of 4 to 8 days. Where indicated, DMEM containing the noted breast cancer cell secretome was used for the first 2 days of adipogenesis induction. Conditioned media was used at a one-to-one ratio with fresh DMEM, plus 10% FBS (final) and differentiation cocktail. To increase the dynamic range and better quantify rescue of adipogenesis using conditioned media from knockout cells, conditioned media collected from MDA-MB-468 sgRNA cells (and controls) was used at 100% (i.e., no fresh DMEM added) in the 3T3-L1 differentiation media. No conditioned media was used past the first 2 days of adipogenesis induction (i.e., in the maintenance media). Where indicated, differentiation media was supplemented with recombinant ZAG (R&D Systems #4764-ZA) or TSP-1 (R&D Systems #3074-TH). No conditioned media was used in the recombinant protein experiments. See Supplementary Table S1 for oligos used to generate 3T3-L1 cell lines depleted of candidate factors.

Mouse primary ASPCs were grown to confluency immediately following isolation in DMEM containing 10% FBS without any passaging. Adipogenesis was induced using DMEM containing 10% FBS and differentiation cocktail consisting of insulin, dexamethasone, and IBMX (as described above). After 3 days of differentiation cocktail, media was changed to DMEM containing 10% FBS and 1 μg/mL insulin. Maintenance media was changed every 2 to 3 days. Breast cancer cell–conditioned media was used during the first 3 days of adipogenesis induction where indicated (i.e., in differentiation media). TGFβ at 2 ng/mL (Miltenyi Biotec, 130-095-067) treatment was used during the first 3 days of adipogenesis induction where indicated.

### Live-cell imaging analysis for lipid accumulation and proliferation

Live imaging for kinetics and end-point quantification of adipogenesis was performed using the IncuCyte Live Cell Analysis Imaging System (Essen Bioscience) with images acquired every 4 hours using the 10× objective. Cells were differentiated as described above and supplemented with 2 µmol/L BODIPY 493/503 (4,4-Difluoro-1,3,5,7,8-Pentamethyl-4-Bora-3a,4a-Diaza-s-Indacene; Thermo, D3922) throughout the adipogenesis time course. Green fluorescence images were acquired every 4 hours using default IncuCyte setting. Total green fluorescence intensity was determined from a green fluorescent mask generated by the IncuCyte Zoom Analysis Software. For end-point analysis, final total green fluorescence intensity values (day 4 or day 5 of differentiation) were normalized to “no differentiation control” and “DMEM control differentiation” (no secretome). Proliferation was assessed using a confluency mask generated by the IncuCyte Zoom Analysis Software using phase images.

### Proteomics analysis

Digestion of in-gel proteins: proteins were reduced with 5 mmol/L DTT for 45 minutes at 60°C and then alkylated with 10 mmol/L IAA for 30 minutes at room temperature in the dark. Trypsin/LysC mixture was added to protein sample in a 1:100 ratio. Proteins were digested for overnight at 38°C. The digestion was quenched by acidification with 1% formic acid to a pH of 2 to 3 and the peptides concentrated *en vacuo* to a final volume of 5 µL.

LC-MS/MS analysis: reversed-phase nano-LC/MS-MS was performed on an UltiMate 3000 RSLCnano system (Dionex) with a PharmaFluidics μPAC micro-chip based trapping column and a 50 cm equivalent PharmaFluidics μPAC micro-chip based column (Pharmafluidics, Ghent, Belgium). Peptide fractions were reconstituted in 100 μL of 0.1% formic acid in water. Five microliters of the samples were injected into the liquid chromatograph. A gradient of reversed-phase buffers (Buffer A: 0.1% formic acid in water; Buffer B: 0.1% formic acid in acetonitrile) at a flow rate of 0.5 mL/minutes was set-up. The LC run lasted for 90 minutes with a starting concentration of 1% buffer B increasing to 28% over the initial 72 minutes with a further increase in concentration to 50% over 18 minutes. A final ramp up to 95% took place over 5 minutes and was held for 10 minutes before ramping down to 1% B over 2 minutes and re-equilibrating for 10 minutes. A QExactive HF (ThermoFisher Scientific) coupled to a Flex nano spray source was employed with the following settings for MS1: resolution 120,000, AGC target 1e5, maximum IT 50 ms, scan range 375 to 1,400 m/z. MS2 settings were: resolution 60,000, AGC target 1e5, maximum IT 100 ms, isolation window 1.2 m/z. Top 15 DDA analysis was performed with NCE set to 32.

Analysis of MS-MS data: Proteome Discoverer (Version 2.4) was used for database searching and protein identification. For these samples, the Uniprot database was searched with Homo sapiens taxonomy selected. The parameters used for the Mascot searches were: no enzyme digest; two missed cleavages; carbamidomethylation of cysteine set as fixed modification; oxidation of methionine, acetylation of n-terminus, amidation of c-terminus, and deamidated of NQ, were set as variable modifications; and the maximum allowed mass deviation was set at 11 ppm.

### Clinical data analysis of human patients with breast cancer

We used cBioPortal (33–35) to analyze the two gene expression data sets, The Cancer Genomic Atlas (TCGA; ref. 36) and Metabric (37–39), of patients with breast cancer. These data sets were chosen because of availability of gene expression data as well as ER, PR, and HER2 status for all patients. All analyses were performed for both data sets combined or individually, groups were classified as all breast cancer, ER-positive breast cancer (ER status positive), or TNBC (ER status negative, HER2 status negative, PR status negative). For ZAG expression, mRNA expression *z*-scores relative to all samples (log microarray) were plotted for each group. For clinical outcome, ZAG/AZGP1 expression [mRNA expression *z*-scores relative to all samples (log microarray)] was binned by quartiles and survival was plotted. Analysis was repeated for each group (all breast cancer, ER-positive breast cancer, TNBC) and the two data sets combined and individually. Multivariate Cox regression analysis was performed using R version 4.3.2 and accounting for therapy (chemo, hormone, radio) and cohort.

### Xenograft studies

Orthotopic xenograft studies were performed in immunodeficient 5-week-old NRG (JAX # 007799) mice. Two independent studies were done weeks apart, and each experimental cohort included total of 10 mice (5 for control cells + 5 for ZAG depleted cells). 5 × 10^6^ cells of MDA-MB-468 sgSafe (control) or MDA-MB-468 sgZAG were injected with Matrigel into the fourth mammary fat pad of female NRG mice. During the course of the study, mice were weighed 1× per week, and tumors were measured in millimeters by length and width 2× per week to calculate the tumor volumes. At the end of the study, all mice from each of the experimental cohorts were euthanized together, and tumors with surrounding adipose tissue were collected, embedded, and stained for analysis.

### Immunofluorescence staining

Tumors and surrounding adipose tissue were paraffin embedded and sectioned by the ARUP Histology Core Laboratory. For deparaffinization of tumor sections, slides were warmed at 70°C for 45 minutes. The slides were then incubated in the following series of solutions at room temperature: 100% xylene (Sigma-Aldrich, 534056) for 15 minutes (two times), 75% xylene and 25% ethanol (Decon Labs, 64-17-5) for 7 minutes, 50% xylene and 50% ethanol for 5 minutes, 25% xylene and 75% ethanol for 5 minutes, 100% ethanol for 5 minutes (two times), 75% ethanol for 5 minutes, 50% ethanol for 5 minutes, 25% ethanol for 5 minutes, then tumor section slides were washed in 100% water for 5 minutes. Heat-induced epitope retrieval was used for the following antibodies: Ki67 (Cell Signaling D35B, 1:200), Alpha-Smooth Muscle Actin (αSMA; Invitrogen, 14-9760-82, 1:200); briefly, 10 mmol/L sodium citrate buffer (10 mmol/L sodium citrate, 0,05% Tween 20, pH 6.0) was warmed to 95°C. Slides were then incubated in sodium citrate buffer for 15 minutes, followed by cooling at room temperature for 20 minutes.

For immunofluorescence staining, slides were washed three times for 5 minutes with PBS at room temperature. Slides were then blocked for 60 minutes with blocking buffer {1% PBSA [1% BSA (Sigma-Aldrich, A7906) in PBS)]} for 60 minutes. Slides were then incubated in 1% PBSA with primary antibody for 60 minutes, followed by washing the sections in 1% PBSA three times for 10 minutes at room temperature. Slides were incubated with secondary antibody (Alexa Fluoro, 1:200 dilution) in 1% PBSA for 1 hour. Slides were then incubated DAPI (1:1,000) for 5 minutes, followed by washing the sections with 1% PBSA three times for 10 minutes. Fluoromount -G (Southern Biotech, 0100-01) was used to mount slides with cover slips. Primary antibodies used were the following: Ki67 (Cell Signaling D35B, 1:200), αSMA (Invitrogen, 14-9760-82, 1:200).

### *In vivo* animal studies

C57Bl/6J mice were purchased from Jackson Laboratory (000664). All animal studies were approved by and were treated in accordance with the University of Utah, Institutional Animal Care and Use Committee guidelines and policies. Male mice between 6 and 8 weeks of age were used for isolation of murine primary ASPCs. Female NRG mice (JAX # 007799) at 5 weeks of age were used for the orthotopic xenograft experiments.

### Statistical analysis

Statistical parameters including the statistical test used, exact value of *n*, what *n* represents, and the distribution and deviation are reported in the figures and corresponding figure legends. Most data are represented as the mean ± standard deviation, data points show independent biological replicates, and the *P* value was determined using unpaired two-tailed Student *t* tests, one-way ANOVA, or two-way ANOVA. Unless otherwise stated, statistical analyses were performed in GraphPad Prism.

### Data availability

Further information and requests for resources and reagents should be directed to and will be fulfilled by the Lead Contact, Keren Hilgendorf (keren.hilgendorf@biochem.utah.edu). All unique/stable reagents generated in this study are available from the Lead Contact with a completed Materials Transfer Agreement. The published article includes all datasets generated during this study.

## Results

### Breast cancer cells secrete factors that modulate adipogenesis

Previous studies have shown that breast cancer cells can transform the white adipose tissue tumor microenvironment to become more tumor supportive. To identify factors secreted by breast cancer cells to regulate the ASPC fate, we obtained 10 distinct human breast cancer cell lines, encompassing all clinical subtypes (Supplementary Fig. S1A). Once breast cancer cell lines were confluent, the growth media was changed to DMEM. Conditioned media containing the breast cancer cell secretome was collected 72 hours later. To study the potential effect of the secretome on adipogenesis, we used a well-characterized, murine cell line model of adipogenesis, 3T3-L1 cells. These cells differentiate uniformly in response to an adipogenic differentiation cocktail containing insulin, dexamethasone, and the cAMP-elevating agent IBMX.

**Figure 1:**
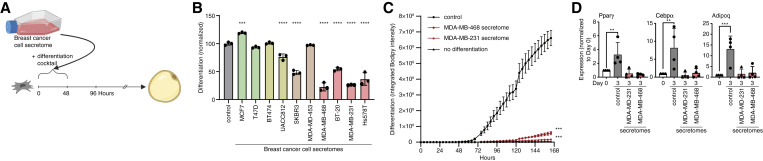
Secretome of human breast cancer cells modulates adipogenesis. **A,** Schematic of experimental workflow. Breast cancer cell secretomes were added only during the first 2 days of 3T3-L1 adipogenesis. **B,** Effect of human breast cancer cell line secretomes on 3T3-L1 adipogenesis. **C** and **D,** The secretome of TNBC cell lines MDA-MB-468 and MDA-MB-231 inhibits the differentiation of primary murine ASPCs as assessed by (**C**) lipid accumulation during adipogenesis and (**D**) expression of adipogenic genes *Pparγ*, *Cebpα*, and *Adipoq*. **B–D,** All data are mean ± SD. Data points show independent biological replicates. *P* values calculated using (**B** and **D**) one-way ANOVA followed by Dunnett’s multiple comparison test or (**C**) two-way ANOVA followed by Šidák’s multiple comparison test. (**, *P* < 0.01; ***, *P* < 0.001; ****, *P* < 0.0001; **A,** Created with BioRender.com.)

3T3-L1 cells were differentiated using breast cancer cell conditioned media mixed in a one-to-one ratio with fresh DMEM and supplemented with serum and adipogenic differentiation cocktail (Fig. 1A). 3T3-L1 adipogenesis was monitored by quantifying intracellular lipid accumulation using a lipophilic, green fluorescent dye BODIPY 493/503, and the IncuCyte live-cell imaging system. This allows for both quantification of intracellular lipid content at the end point of the adipogenesis protocol and kinetic analysis of intracellular lipid accumulation during adipogenesis. Strikingly, the secretome of most breast cancer cells significantly modulated 3T3-L1 adipogenesis (Fig. 1B; Supplementary Fig. S1B and S1C). In particular, the secretomes of all TNBC cells tested severely inhibited 3T3-L1 adipogenesis (Fig. 1B; Supplementary Fig. S1B and S1C). Only the secretome of the luminal A breast cancer cell line MCF7 promotes 3T3-L1 adipogenesis. The secretomes of luminal B breast cancer cell lines and HER2-positive breast cancer cell lines had modest or no effects on 3T3-L1 adipogenesis.

Given the dramatic and uniform effect of TNBC secretomes on 3T3-L1 adipogenesis, we next assessed its effect on primary ASPCs freshly isolated from the inguinal white adipose tissue of C57Bl/6 mice. Remarkably, the presence of TNBC secretome completely abrogated adipogenesis as assessed by intracellular lipid content (Fig. 1C; Supplementary Fig. S1D). Similarly, the presence of TNBC secretome inhibited the expression and activation of known adipogenic transcription factors and regulators as assessed by quantitative real-time PCR (Fig. 1D) or using a PPARγ reporter cell line (Supplementary Fig. S1E; ref. 40). Thus, all TNBC cell lines tested secrete one or more paracrine factors that potently inhibit the differentiation of ASPCs.

### ZAG is necessary and sufficient for TNBC-mediated inhibition of adipogenesis

**Figure 2:**
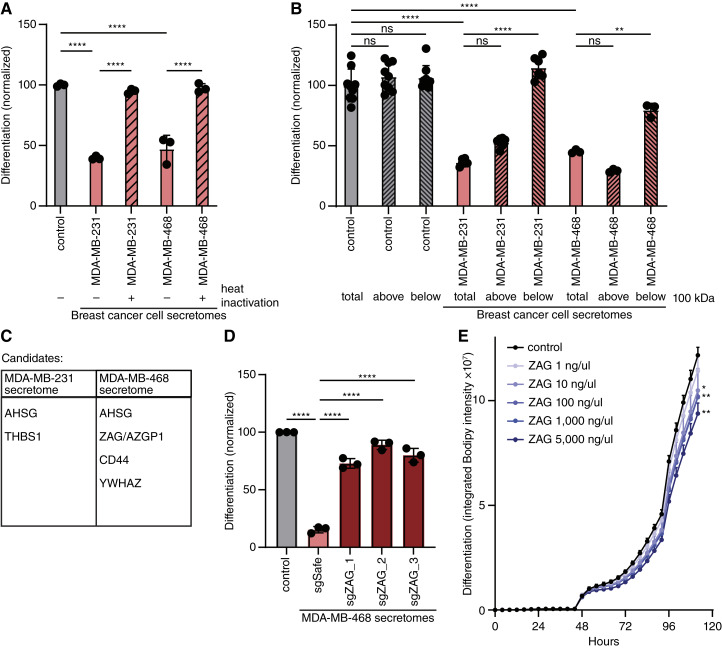
The glycoprotein ZAG inhibits adipogenesis. **A,** Inhibition of 3T3-L1 adipogenesis is rescued with heat inactivation of the secretomes of MDA-MB-468 and MDA-MB-231 cells. **B,** The antiadipogenic factor of the MDA-MB-468 and MDA-MB-231 secretomes (total) is retained in the supernatant (top) fraction following centrifugation using a spin column with a molecular weight cut-off of 100,000 Da. **C,** Candidate antiadipogenic factors identified in the secretomes of MDA-MB-468 and MDA-MB-231 cells. **D,** The secretome of MDA-MB-468 cells depleted of ZAG does not inhibit 3T3-L1 adipogenesis. **E,** Supplementing 3T3-L1 cells with recombinant ZAG during the first 2 days of adipogenesis is sufficient to inhibit adipogenesis. **A–E,** All data are mean ± SD. Data points show independent biological replicates. *P* values calculated using one-way ANOVA followed by (**A** and **B**) Tukey’s multiple comparison test or (**D**) Dunnett’s multiple comparison test, or (**E**) two-way ANOVA followed by Šidák’s multiple comparison test. (ns, non-significant; *, *P* < 0.05; **, *P* < 0.01; ****, *P* < 0.0001.)

To identify the antiadipogenic factor secreted by TNBC cells, we first sought to determine the class of macromolecule. Specifically, we heat inactivated the secretome of two TNBC cell lines, MDA-MB-231 and MDA-MB-468 cells, to denature heat-labile proteins. Heat inactivation completely rescued 3T3-L1 adipogenesis (Fig. 2A; Supplementary Fig. S2A). Thus, the secretome of these two TNBC cell lines contains one or more heat-labile proteins required for the inhibition of adipogenesis. Next, we sought to enrich for a fraction containing this antiadipogenic protein(s) using centrifugal filter columns. TNBC secretomes were subjected to spin columns with a molecular weight cut-off of 100,000 Da. 3T3-L1 cells were then differentiated in the presence of either the filtrate or supernatant. The ability of the TNBC secretome to inhibit 3T3-L1 adipogenesis was only retained in the supernatant (Fig. 2B; Supplementary Fig. S2B). Of note, the conditioned media containing TNBC secretome was collected in defined DMEM only as described above (i.e., no serum), which does not contain any proteins greater than 100,000 Da in size. Thus, our initial biochemical characterization of the TNBC secretome likely yielded a fraction relatively depleted of proteins but highly enriched for the unknown antiadipogenic protein(s) of interest.

We next performed protein mass spectrometry on the spin column supernatants of three independent secretomes collected from two TNBC cell lines, MDA-MB-231 and MDA-MB-468 cells (Supplementary Table S2). After excluding abundant intracellular protein contaminants, this analysis yielded five candidate antiadipogenic proteins, only one of which was found in the secretomes of both TNBC cell lines (Fig. 2C). To identify which of these candidate antiadipogenic proteins is required in the TNBC secretome to inhibit 3T3-L1 adipogenesis, we next individually knocked out each candidate in both TNBC cell lines using Crispr/Cas9. Specifically, we depleted ASHG, ZAG (also known as AZGP1), CD44, or YWHAZ using three distinct sgRNA each in both MDA-MB-468 and MDA-MB-231 cell lines. Remarkably, the secretome of all three MDA-MB-468 cell lines and all three MDA-MB-231 cell lines depleted for ZAG rescued 3T3-L1 adipogenesis (Fig. 2D; Supplementary Fig. S2C–G). Thus, the presence of ZAG is required for the MDA-MB-468 and the MDA-MB-231 secretomes to inhibit 3T3-L1 adipogenesis. Loss of AHSG, CD44, or YWHAZ in either TNBC cell line did not rescue adipogenesis, nor was recombinant TSP-1 sufficient to inhibit 3T3-L1 adipogenesis (Supplementary Fig. S3A–C). In contrast, the presence of recombinant ZAG during the first 2 days of 3T3-L1 adipogenesis is sufficient to inhibit adipogenesis in a dose-dependent manner, albeit not as efficiently as the TNBC secretome (Fig. 2E; Supplementary Fig. S3D). Together, these data show that ZAG is both necessary and sufficient for TNBC cells to inhibit adipogenesis. We note that we were only able to identify ZAG by immunoprecipitation in the secretome of MDA-MB-468 cells and not in the secretome of MDA-MB-231 cells (Supplementary Fig. S4A). As the MDA-MB-231 secretome potently inhibits adipogenesis and this is rescued by ZAG depletion, we postulate that ZAG secreted by MDA-MB-231 may be differentially modified or unable to be detected due to other technical limitations. We, therefore, used MDA-MB-468 cells with and without validated ZAG depletion to study the role of ZAG in tumorigenesis.

### ZAG is expressed by a subset of TNBC cells and linked to poor prognosis

ZAG is primarily secreted by mature adipocytes and promotes adipocyte lipolysis to enable energy homeostasis (41). Plasma levels in healthy adults are 18 to 30 mg/dL, and this is decreased in obese patients and mice (42). Of note, the level of ZAG present in the MDA-MB-468 secretome capable of inhibiting adipogenesis is therefore well within the physiologically relevant range (Supplementary Fig. S4B). Interestingly, ZAG expression is increased in the adipose tissue of patients with cancer cachexia, and ZAG is identical to the lipid-mobilizing factor purified from urine of patients with cancer cachexia (43). Moreover, ZAG has also been shown to be expressed by several types of malignant tumors, including breast, prostate, and lung (44–46).

**Figure 3:**
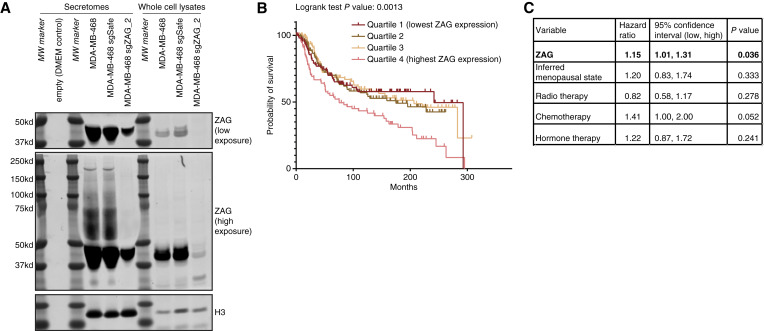
ZAG is secreted by TNBC cells and linked to poor prognosis. **A,** MDA-MB-468-secreted ZAG is highly modified (lanes 4, 5). These high molecular weight species were not observed in the secretome of ZAG depleted cells (lane 6), DMEM-only media control (lane 2), or in whole cell MDA-MB-468 lysates (lanes 8, 9). Histone 3 used as lysate loading control and also found in secretome, likely due to sparse cell death. **B,** TNBC outcome of combined Metabric and TCGA data sets stratified by ZAG expression. **C,** Table of multivariable Cox proportional hazards model shows ZAG expression is independent risk factor for poor survival in TNBC after adjusted for menopause status and therapy.

ZAG secreted by adipocytes or cancer cells is known to be highly glycosylated (47). Accordingly, we observed the presence of high molecular weight species in the secretome of MDA-MB-468 parental cells and control cells expressing a sgRNA targeting a safe locus (Fig. 3A). These high molecular weight species are only present in the secretome, not the cell lysate and are greatly depleted in a pool of MDA-MB-468 cells depleted of ZAG, confirming the specificity of the antibody.

Next, we analyzed two large gene expression data sets from patients with breast cancer [TCGA (36) and Metabric (37–39)] using cBioPortal (33–35). ZAG expression in TNBC cells is highly variable (Supplementary Fig. S4C). Remarkably, stratifying clinical outcome of all TNBC by ZAG expression shows that patients with TNBC expressing ZAG at high levels have significantly worse prognosis (Fig. 3B; Supplementary Fig. S4D). We next calculated the hazard ratio for ZAG. We show that ZAG expression is a significant predictor for poor survival in TNBC both in a univariate analysis and after adjusting for menopause status, therapy, and batch effects. Specifically, each one-unit increase in ZAG expression is linked to a 15% increase in risk (Fig. 3C). This argues that the ZAG, secreted by TNBC cells and capable of inhibiting adipogenesis, promotes tumorigenesis. Interestingly, the mean expression of ZAG is lower in TNBC than in ER-positive breast cancer (Supplementary Fig. S4C). However, stratifying clinical outcome of ER-positive breast cancer by ZAG expression shows no correlation with poor prognosis (Supplementary Fig. S4E and S4F). Thus, TNBC-secreted ZAG is functionally distinct from ZAG secreted by other clinical subtypes in promoting tumorigenesis.

### ZAG promotes TNBC tumor growth and induces the expression of fibrotic genes in ASPCs

**Figure 4:**
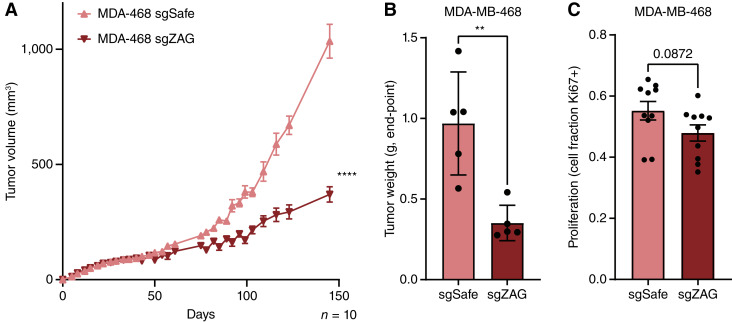
ZAG is important for MDA-MB-468 tumorigenesis. **A** and **B,** Depletion of ZAG inhibits xenograft growth of MDA-MB-468 cells as determined by (**A**) tumor volume measurements over time or (**B**) weight of excised tumor at the end point. **C,** MDA-MB-468 cells depleted for ZAG show a trend of decreased proliferation *in vivo*. **A–C,** Data are mean ± SEM. Data points and *n* are independent mice. *P* values calculated using (**A**) two-way ANOVA followed or (**B** and **C**) Student *t* test. (**, *P* < 0.01; ****, *P* < 0.0001.)

To explore the role of ZAG in MDA-MB-468 tumorigenesis, we next injected control or ZAG-depleted cells into the fourth mammary fat pad of female NRG mice. ZAG depletion potently inhibits orthotopic xenograft growth (Fig. 4A; Supplementary Fig. S5A). Consistent with decreased tumor burden at the experimental end point (Fig. 4B; Supplementary Fig. S5B), loss of ZAG resulted in decreased proliferation of MDA-MB-468 cells *in vivo*, though this difference did not reach significance (Fig. 4C). Importantly, these effects were not cell intrinsic, as loss of ZAG does not affect MDA-MB-468 proliferation *ex vivo* (Supplementary Fig. S5C).

**Figure 5:**
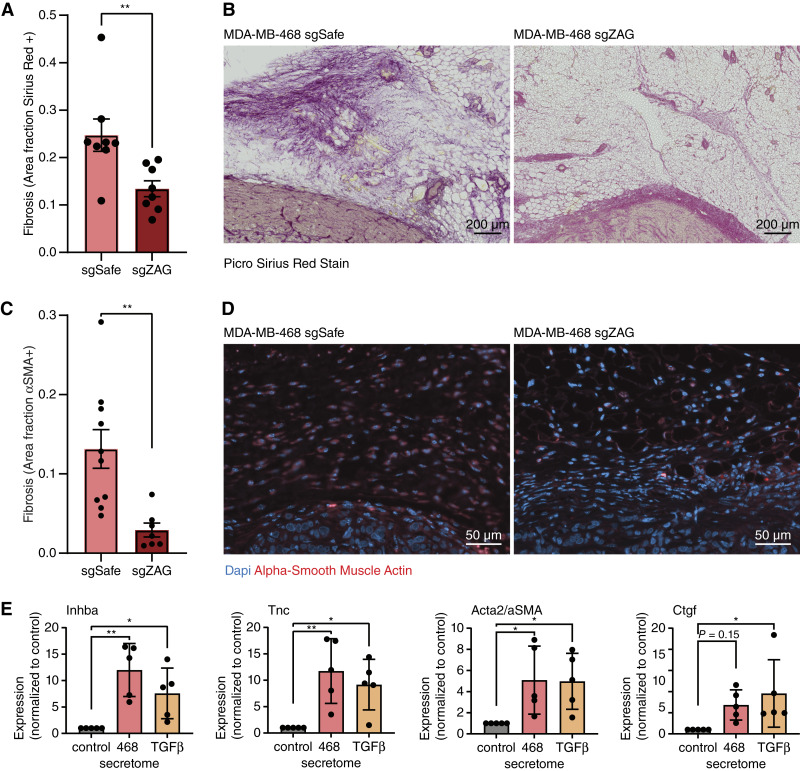
TNBC-secreted ZAG promotes fibrosis in the surrounding adipose tissue. **A–D,** The adipose tissue microenvironment of MDA-MB-468 xenografts depleted for ZAG is less fibrotic. **A,** Quantification and (**B**) representative image of Picro Sirius Red staining. **C,** Quantification and (**D**) representative image of αSMA staining. Sirius Red and αSMA staining was quantified in the region of interest defined as within 2 mm surrounding the tumor. **E,** The secretome of MDA-MB-468 cells promotes the expression of fibrotic genes *Inhba*, *Tnc*, *Acta* (*αSma*), and *Ctgf* in differentiating primary murine ASPCs (day 3 of adipogenic differentiation). TGFβ treatment of ASPCs for 3 days shows similar induced expression of fibrotic genes. **A** and **C,** Data are mean ± SEM. Data points are quantification of the staining of sections from 10 independent mice. *P* values calculated using Student *t* test. **E,** Data are mean ± SD. Data points show independent biological replicates from separate ASPC isolations. *P* values calculated using one-way ANOVA followed by Dunnett’s multiple comparison test. (ns, non-significant; *, *P* < 0.05; **, *P* < 0.01.)

Given the known role of ZAG in inducing lipolysis, we first quantified the size of tumor-adjacent adipocytes. Loss of ZAG did not affect the size of cancer-associated adipocytes (Supplementary Fig. S6A–C). As we had initially identified ZAG as a factor that inhibits ASPC adipogenesis, we next hypothesized that it may instead promote the transdifferentiation of ASPCs into cancer-associated fibroblasts. Remarkably, staining the adipose tissue tumor microenvironment for markers of fibrosis uncovered dramatic differences. Specifically, ZAG depletion in MDA-MB-468 cells caused a significant reduction in both Picro Sirius Red staining and αSMA staining in the surrounding adipose tissue (Fig. 5A–D). We therefore assayed for the induced expression of fibrotic markers in primary murine ASPCs differentiated in the presence of MDA-MB-468 secretome. Consistent with the increased fibrosis observed in the adipose tissue tumor microenvironment, we show that exposing primary ASPCs to the secretome of MDA-MB-468 cells promotes the expression of canonical fibrosis genes (Fig. 5E). Thus, we identified a glycoprotein, ZAG, that is secreted by a subset of TNBC cells and may promote tumorigenesis by inhibiting adipogenesis and instead promoting the transdifferentiation of ASPCs into cancer-associated fibroblasts.

## Discussion

The adipose tissue tumor microenvironment is critical for breast cancer growth, and breast cancer cells are known to transform the surrounding adipose tissue into a more tumor-supportive microenvironment. Here, we screened the secretomes of 10 different human breast cancer cell lines, encompassing all clinical subtypes, for the ability to regulate ASPC fate. We identified the cachexia-associated glycoprotein, ZAG, which promotes TNBC growth and fibrosis in the adipose tissue tumor microenvironment. High ZAG expression in TNBC is linked to poor prognosis. Together, these data suggest that targeting ZAG in TNBC may have therapeutic potential.

In healthy adults, ZAG is primarily secreted by mature adipocytes to promote lipid mobilization in an autocrine or paracrine manner (41). Previous studies have shown that ZAG is also expressed by malignant tumor cells, including breast cancer cells (45). Xenograft studies using ZAG-overexpressing cells show shrunken, beige fat-like adipocytes (48). Overexpression of ZAG in 3T3-L1 adipocytes inhibits the expression of lipogenic genes, and stimulates adipocyte lipolysis and beiging through increased expression of lipases, including adipose triglyceride lipase (ATGL) and hormone-sensitive lipase (HSL), and beige fat-specific markers (49). Here, we identified a novel function of ZAG in regulating ASPC fate in adipose tissue. This discovery is distinct and separate from lipogenesis/lipolysis in mature adipocytes, as we observe a decrease in the expression of canonical adipogenic transcription factors when differentiation of ASPCs is initiated in the presence of ZAG. Further, we do not observe any changes in adipocyte size in the tumor microenvironment in xenograft studies using ZAG-depleted breast cancer cells. We note that the timeframe of our *in vivo* experiments precludes changes in adipocyte size due to alterations in the rate of adipogenesis (50, 51). Together, this suggests that TNBC cells may use ZAG to remodel the adjacent adipose tissue to both increase stiffness by targeting ASPCs and/or increase lipid fuels by targeting mature adipocytes. We do not know if ZAG also modulates intratumoral fibrosis via a mechanism that is independent of adipose tissue microenvironment-resident ASPCs.

TNBC-secreted ZAG promotes the trans-differentiation of ASPCs into myofibroblast-like cells, and the accumulation of myofibroblasts in the tumor-adjacent stroma has previously been linked to poor prognosis and to increased collagen deposition, collagen cross-linking, and tissue stiffness in the tumor microenvironment of ovarian cancer (52), breast cancer (53), and colorectal cancer (54). Mechanistically, cancer-associated myofibroblasts secrete and crosslink collagen (55). Myofibroblasts are characterized by the high expression of αSMA resulting in enhanced contractility (56). This contractility promotes the excessive deposition of collagen and other extracellular matrix components by myofibroblasts and surrounding cells, resulting in tissue stiffening (57). Finally, the stiffening of the surrounding stroma is thought to enhance several steps of tumorigenesis, including cancer initiation and progression (24, 53, 58), proliferation (59), and metastasis (60, 61).

Here, we show that high ZAG expression by TNBC is associated with poor prognosis. However, our comprehensive analysis of breast cancer patient gene expression data did not reveal any unidirectional association between ZAG expression and prognosis when all clinical subtypes of breast cancer were combined. Elevated ZAG expression may even be associated with better prognosis in ER-positive breast cancer**.** Interestingly, while ZAG expression is significantly elevated in breast cancer tissue compared to normal breast tissue from healthy women, previous studies have linked ZAG expression in breast cancer with both worse and better prognoses (45, 62–64). The clinical subtype of breast cancer was not considered in most of these studies, potentially accounting for these contradictory observations. Mechanistically, depletion of ZAG in two human breast cancer cell lines, MCF7 and MDA-MB-231, was shown to inhibit proliferation, migration, and invasion *ex vivo*, and tumor growth *in vivo* (63). Recombinant ZAG was also shown to promote the proliferation of MCF7 and MDA-MB-231 cells (65). We did not observe a cell-intrinsic proliferation defect in our cells when ZAG was depleted (Supplementary Fig. S4C). Instead, we showed that recombinant ZAG and TNBC-secreted ZAG inhibited adipogenesis and promoted adipose tissue fibrosis and tumor growth.

Beyond breast cancer, ZAG expression is significantly elevated in colorectal cancer compared to normal tissue, and high ZAG expression or elevated serum ZAG levels are linked with poor prognosis in colorectal cancer (66). ZAG expression is further elevated in colorectal cancer with liver metastasis compared to colorectal cancer without distant metastasis, and ZAG was shown to promote epithelial-to-mesenchymal trans-differentiation and modulate the expression adhesion proteins (67). In contrast, reduced ZAG expression is linked to poor prognosis of prostate cancer, gastric cancer, and hepatocellular carcinoma (68–70). ZAG has also been shown to suppress the growth of oral and pancreatic cancer by downregulating cyclin-dependent kinase 1 gene and inducing mesenchymal-to-epithelial transdifferentiation, respectively (71, 72). ZAG also inhibits fibrosis in the kidney and heart (73). Together, these findings reveal that ZAG exhibits distinct and often opposing functions depending on the cellular context of its expression.

We propose that ZAG secreted by a subset of TNBC is functionally distinct from ZAG secreted by ER-positive breast cancer. Specifically, we show here that TNBC-secreted ZAG promotes the trans-differentiation of ASPCs into cancer-associated fibroblasts. We do not yet know how ZAG secreted by other breast cancer subtypes regulates ASPC fate. How could TNBC-secreted ZAG have a distinct function? One possibility is that ZAG, a highly glycosylated protein, is differentially glycosylated by TNBC cells, and that only the TNBC-specific glycosylation pattern enables ZAG to regulate ASPC fate. Of note, ZAG is abundant in human saliva and a potential biomarker for lung cancer (74); LC-MS/MS analysis of salivary ZAG identified 22 glycan structures, five of which were unique to samples collected from lung cancer patients (47). We do not yet know the glycan structure of TNBC-secreted ZAG. Alternatively, other paracrine factors secreted by TNBC cells may be required for ZAG to promote ASPC trans-differentiation and fibrosis. We note that either model may explain why the recombinant ZAG used in this study was not as efficient at inhibiting adipogenesis as TNBC-secreted ZAG. Future investigation will establish if and how TNBC-secreted ZAG differs from ZAG secreted by other sources in terms of post-translational modifications or available interacting proteins. As ZAG expression is linked with poor or better prognosis across several types of cancer (67–70), there is also a need to broadly interrogate the function of ZAG sourced from a multitude of types of cancers.

Even within TNBC, we found significant heterogeneity in ZAG expression. As TNBC is defined as a lack of markers, this likely reflects the inherent histological and molecular heterogeneity of cancers classified as TNBC. Nonetheless, all four TNBC secretomes tested inhibited adipogenesis, and our follow-up investigation on two of these cell lines, MDA-MB-468 and MDA-MB-231, revealed that ZAG is required for this, though we were only able to validate ZAG expression in MDA-MB-468 cells. One recent study showed that ZAG expression is directly regulated by androgen signaling, and that ZAG is specifically secreted by androgen-responsive TNBC, patient-derived xenograft models (75). Thus, specific subtypes within TNBC may modulate the tumor microenvironment via secreted ZAG.

Finally, obesity is a known risk factor for breast cancer, including TNBC. Understanding the underlying mechanisms is complicated by the multifactorial effects of obesity, such as alterations to the cellular composition and morphology of adipose tissue, hyperglycemia, hyperinsulinemia, and hyperlipidemia. Obesity is linked to increased fibrosis in adipose tissue, which is thought to promote breast cancer cell growth. We do not yet know how obesity affects ZAG expression by TNBC, ZAG-dependent remodeling of the adipose tissue tumor microenvironment, and ZAG expression–linked patient prognosis.

## Supplementary Material

Figure S1 Supplemental Figure and Figure Legend 1

Figure S2 Supplemental Figure and Figure Legend 2

Figure S3 Supplemental Figure and Figure Legend 3

Figure S4 Supplemental Figure and Figure Legend 4

Figure S5 Supplemental Figure and Figure Legend 5

Figure S6 Supplemental Figure and Figure Legend 6

Table S2 Supplemental Table 2: Secretome proteomics

Table S1 Supplemental Table 1: Primers used

Supplementary Methods Additional Materials and Methods
