# Supplementary material for: Zinc-alpha-2-glycoprotein Secreted by Triple-Negative Breast Cancer Promotes Peritumoral Fibrosis
Source: Cancer Res Commun. 2024 Jul 5;4(7):1655–66. doi: 10.1158/2767-9764.CRC-24-0218 (PMC11224648; doi:10.1158/2767-9764.CRC-24-0218)
Supplement: Figure S1 — Supplemental Figure and Figure Legend 1 [file crc-24-0218_figure_s1_suppsf1.pdf]

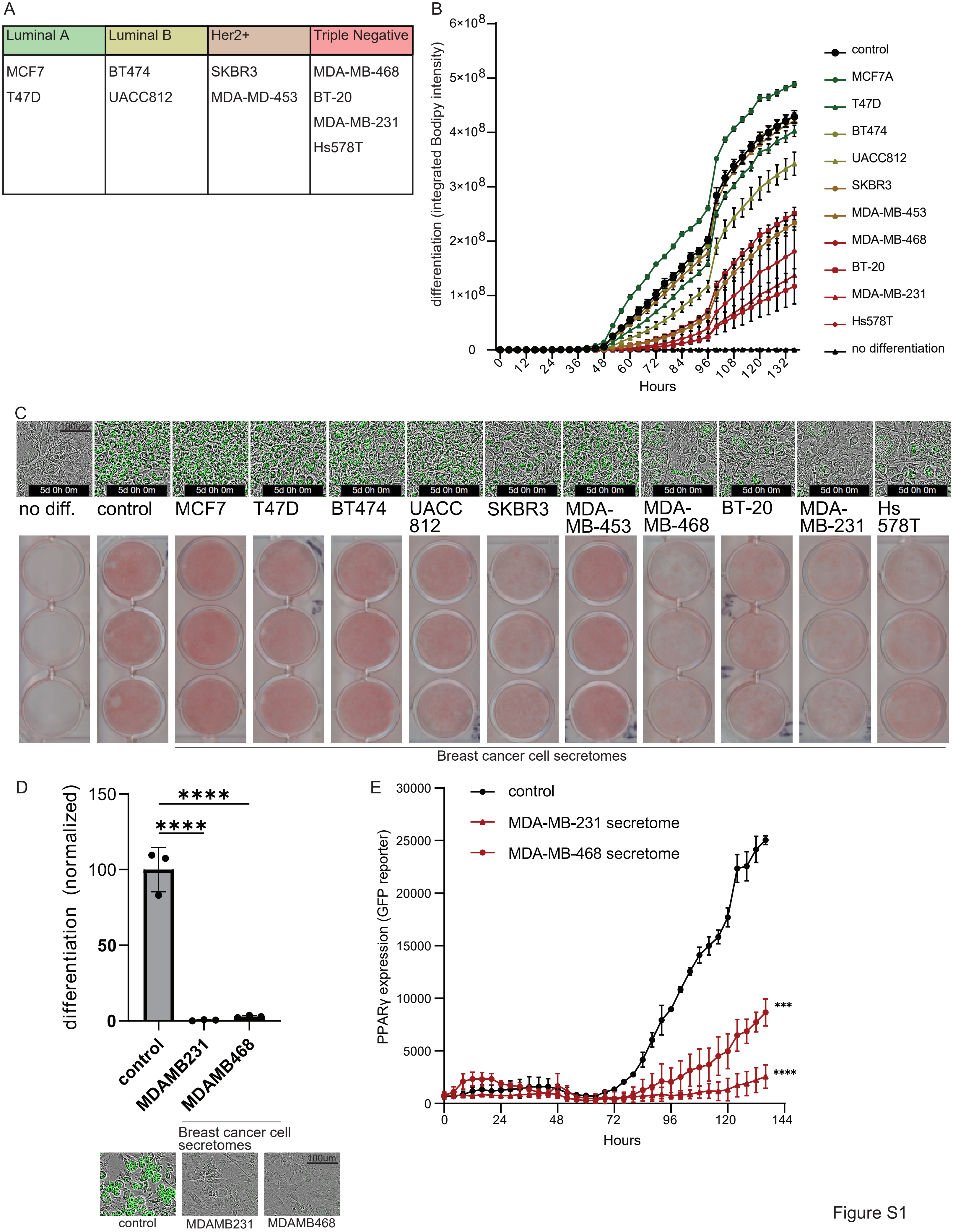
A
Luminal A
Luminal B
Her2+
Triple Negative
MCF7
T47D
BT474
UACC812
SKBR3
MDA-MD-453
MDA-MB-468
BT-20
MDA-MB-231
Hs578T
B
differentiation (integrated Bodipy intensity)
Hours
control
MCF7A
T47D
BT474
UACC812
SKBR3
MDA-MB-453
MDA-MB-468
BT-20
MDA-MB-231
Hs578T
no differentiation
C
100um
5d 0h 0m
no diff.
control
MCF7
T47D
BT474
UACC 812
SKBR3
MDA-MB-453
MDA-MB-468
BT-20
MDA-MB-231
Hs 578T
Breast cancer cell secretomes
D
differentiation (normalized)
****
****
control
MDAMB231
MDAMB468
Breast cancer cell secretomes
100um
control
MDAMB231
MDAMB468
E
PPARγ expression (GFP reporter)
control
MDA-MB-231 secretome
MDA-MB-468 secretome
***
****
Hours

Figure S1

Figure S1: related to Figure 1. Breast cancer line secretomes modulate adipogenesis.

(A) Table of human breast cancer cell lines used, categorized according to clinical subtype noted by the American Type Culture Collection. (B) Time course of lipid accumulation as assessed by total integrated green fluorescent BODIPY intensity during 3T3-L1 adipogenesis. See Figure 1B for endpoint analysis of adipogenesis. The secretomes of human breast cancer cell lines modulates adipogenesis. (C) Images showing total lipid accumulation of 3T3-L1 cells differentiated in the presence of human breast cancer cell line secretome on Day 5 of differentiation using BODIPY staining on live cells (top row) or at experimental endpoint using Oil Red O staining (bottom). (D) End-point analysis of total lipid accumulation as a measure of adipogenesis. See Figure 1C for time course analysis of lipid accumulation during adipogenesis. The secretomes of TNBC cell lines MDA-MB-468 and MDA-MB-231 inhibit the differentiation of primary murine ASPCs as assessed by total integrated green fluorescent BODIPY intensity at the end-point of 3T3-L1 adipogenesis and normalized to DMEM control (no secretome). Images showing BODIPY staining at end-point. (E) A PPARγ-T2A-GFP knockin reporter shows decreased expression of PPARγ in 3T3-L1 cells differentiated in the presence of the secretome of MDA-MB-468 and MDA-MB-231 cells. (B) Data are represented as mean ± SD. (D) Data are represented as mean ± SD. p-values calculated using one-way ANOVA followed by Dunnett's multiple comparison test (****<0.0001). (E) Data are represented as mean ± SD. p-values calculated using two-way ANOVA followed by Šidák's multiple comparison test (***<0.001, ****<0.0001)
