# Supplementary material for: Zinc-alpha-2-glycoprotein Secreted by Triple-Negative Breast Cancer Promotes Peritumoral Fibrosis
Source: Cancer Res Commun. 2024 Jul 5;4(7):1655–66. doi: 10.1158/2767-9764.CRC-24-0218 (PMC11224648; doi:10.1158/2767-9764.CRC-24-0218)
Supplement: Figure S2 — Supplemental Figure and Figure Legend 2 [file crc-24-0218_figure_s2_suppsf2.pdf]

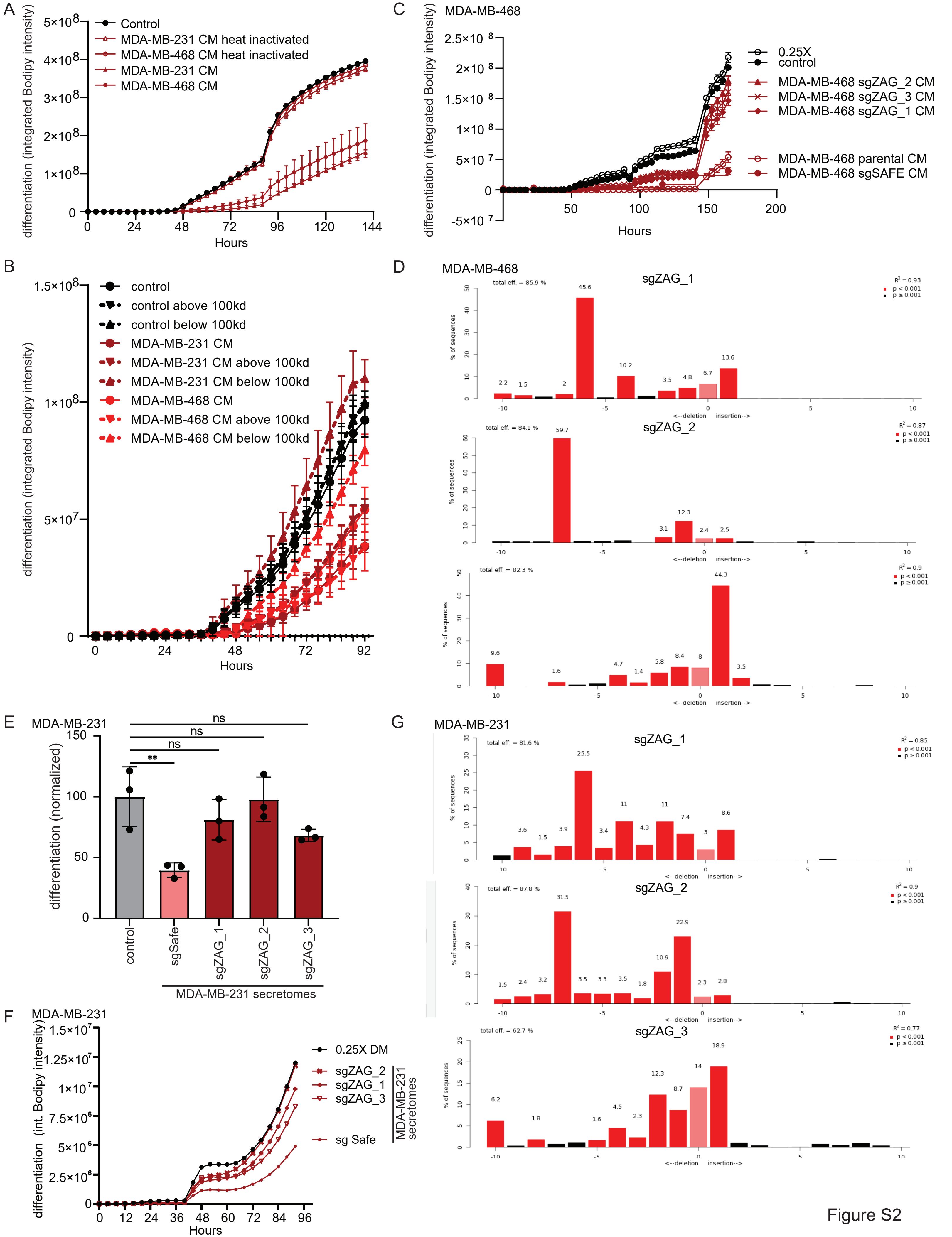
A
Control
MDA-MB-231 CM heat inactivated
MDA-MB-468 CM heat inactivated
MDA-MB-231 CM
MDA-MB-468 CM
differentiation (integrated Bodipy intensity)
Hours
B
control
control above 100kd
control below 100kd
MDA-MB-231 CM
MDA-MB-231 CM above 100kd
MDA-MB-231 CM below 100kd
MDA-MB-468 CM
MDA-MB-468 CM above 100kd
MDA-MB-468 CM below 100kd
differentiation (integrated Bodipy intensity)
Hours
C
MDA-MB-468
0.25X
control
MDA-MB-468 sgZAG_2 CM
MDA-MB-468 sgZAG_3 CM
MDA-MB-468 sgZAG_1 CM
MDA-MB-468 parental CM
MDA-MB-468 sgSAFE CM
differentiation (integrated Bodipy intensity)
Hours
D
MDA-MB-468
sgZAG_1
total eff. = 85.9 %
sgZAG_2
total eff. = 84.1 %
total eff. = 82.3 %
% of sequences
<--deletion insertion-->
E
MDA-MB-231
differentiation (normalized)
ns
ns
ns
**
control
sgSafe
sgZAG_1
sgZAG_2
sgZAG_3
MDA-MB-231 secretomes
F
MDA-MB-231
differentiation (int. Bodipy intensity)
0.25X DM
sgZAG_2
sgZAG_1
sgZAG_3
sg Safe
MDA-MB-231 secretomes
Hours
G
MDA-MB-231
sgZAG_1
total eff. = 81.6 %
sgZAG_2
total eff. = 87.8 %
sgZAG_3
total eff. = 62.7 %
% of sequences
<--deletion insertion-->

Figure S2

Figure S2: related to Figure 2. Identification of ZAG in the TNBC secretome as an anti-adipogenic factor.

(A) Time course of lipid accumulation. See Figure 2A for end-point adipogenesis analysis. Heat-inactivated MDA-MB-468 or MDA-MB-231 secretomes do not inhibit 3T3-L1 adipogenesis. (B)Time course analysis of lipid accumulation. See Figure 2B for end-point adipogenesis analysis. 3T3-L1 cells were differentiated in the presence of unfractionated MDA-MB-468 or MDA-MB-231 secretomes (CM), the supernatant following centrifugal filtration (above 100kd), or the filtrate (below 100kd). The top fractions of fractionated MDA-MB-468 or MDA-MB-231 secretomes inhibit 3T3-L1 adipogenesis. Of note, spin columns do not accurately fractionate all proteins according to indicated molecular weight due to differential folding and/or posttranslational modifications, and cut-off values should not be regarded as definitive values of molecular weights for unknown proteins. (C) Time course of lipid accumulation. See Figure 2D for end-point adipogenesis analysis. ZAG is required for the MDA-MB-468 secretome to inhibit 3T3-L1 adipogenesis. (D) TIDE analysis showing genomic alterations in the three engineered MDA-MB-468 sgZAG cell lines. Each cell line was infected with the one sgRNA (see Table S2) and cell lines are pools of recombination (i.e., not clonal cell lines). (E-F) The secretome of MDA-MB-231 cells depleted of ZAG does not inhibit 3T3-L1 adipogenesis as shown (E) in an end-point analysis and (F) in a time course of lipid accumulation. (G) TIDE analysis showing genomic alterations in the three engineered MDA-MB-231 sgZAG cell lines. Each cell line was infected with the one sgRNA (see Table S2) and cell lines are pools of recombination (i.e., not clonal cell lines). (A-C, E-F) Data are represented as mean ± SD. (E) p-values calculated using one-way ANOVA followed by Dunnett's multiple comparison test. (ns is non-significant; **<0.01).
