# Supplementary material for: Zinc-alpha-2-glycoprotein Secreted by Triple-Negative Breast Cancer Promotes Peritumoral Fibrosis
Source: Cancer Res Commun. 2024 Jul 5;4(7):1655–66. doi: 10.1158/2767-9764.CRC-24-0218 (PMC11224648; doi:10.1158/2767-9764.CRC-24-0218)
Supplement: Figure S3 — Supplemental Figure and Figure Legend 3 [file crc-24-0218_figure_s3_suppsf3.pdf]

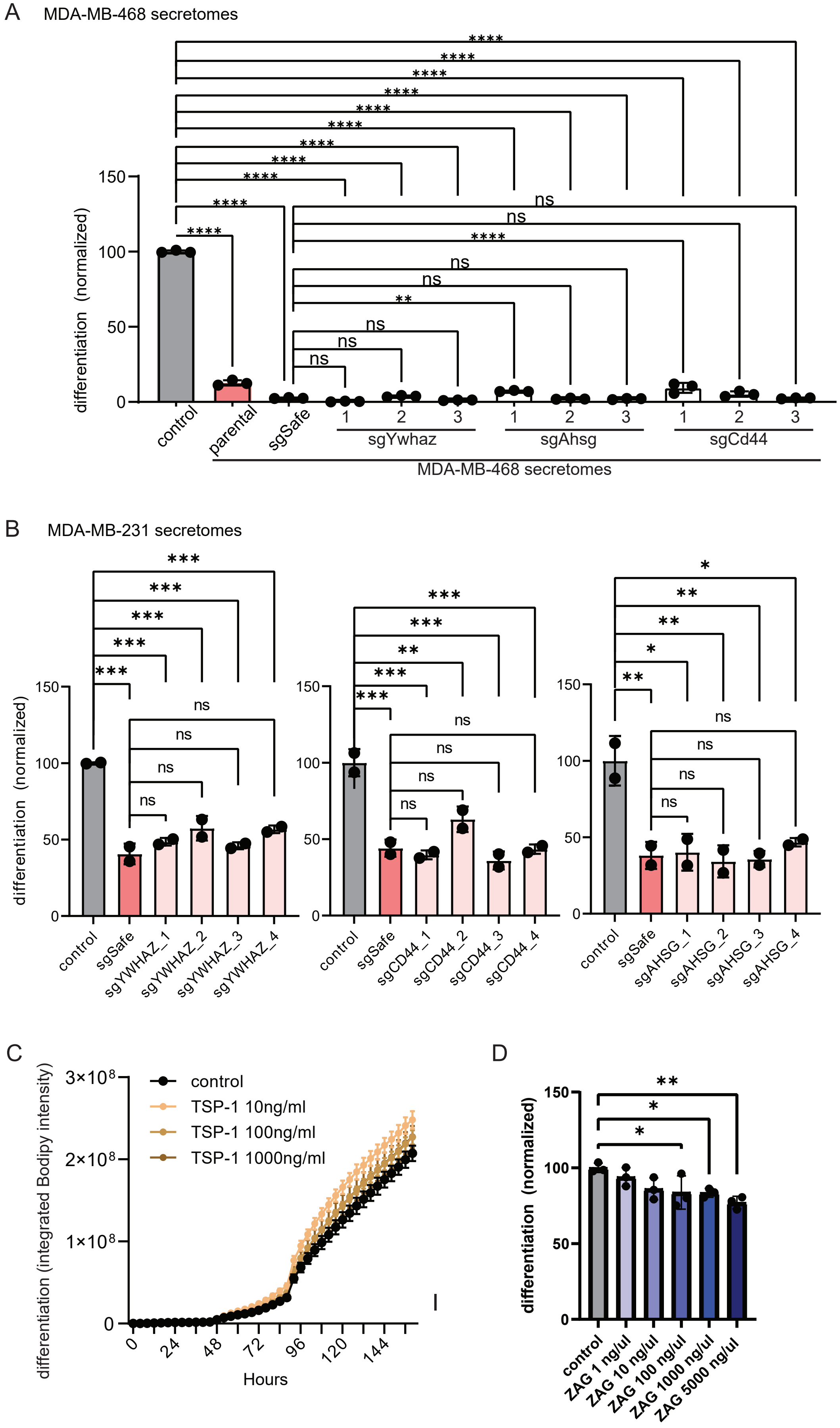
A
MDA-MB-468 secretomes
differentiation (normalized)
control
parental
sgSafe
sgYwhaz
sgAhsg
sgCd44
MDA-MB-468 secretomes
B
MDA-MB-231 secretomes
differentiation (normalized)
control
sgSafe
sgYWHAZ_1
sgYWHAZ_2
sgYWHAZ_3
sgYWHAZ_4
sgCD44_1
sgCD44_2
sgCD44_3
sgCD44_4
sgAHSG_1
sgAHSG_2
sgAHSG_3
sgAHSG_4
C
differentiation (integrated Bodipy intensity)
control
TSP-1 10ng/ml
TSP-1 100ng/ml
TSP-1 1000ng/ml
Hours
D
differentiation (normalized)
control
ZAG 1 ng/ul
ZAG 10 ng/ul
ZAG 100 ng/ul
ZAG 1000 ng/ul
ZAG 5000 ng/ul

Figure S3

Figure S3: related to Figure 2. Screening secretome for anti-adipogenic factors.

(A) The secretomes of MDA-MB-468 cells lacking YWHAZ, AHSG, or CD44 retain the ability to inhibit 3T3-L1 adipogenesis, and the presence of these candidate proteins in the secretome is not required to inhibit adipogenesis. Three distinct cell lines were generated per candidate factor. MDA-MB-468 parental cells are uninfected, MDA-MB-468 sgSafe cells are Cas9-BFP expressing cells infected with an sgRNA targeting a safe genomic locus. (B) Depletion of YWHAZ, AHSG, or CD44 in the secretome of MDA-MB-231 cells does not rescue the inhibition of adipogenesis. Four distinct cell lines were generated per candidate factor. (C) Time course of lipid accumulation of 3T3-L1 cells supplemented with TSP-1 (THBS1) during first 2 days of adipogenesis. TSP-1 does not modulate 3T3-L1 adipogenesis. (D) Adipogenesis endpoint analysis of Figure 2E. Mouse myeloma NS0 cell line-derived human ZAG protein inhibits 3T3-L1 adipogenesis in a dose-dependent manner. (A, B, D) Data are represented as mean ± SD. p-values calculated using one-way ANOVA followed by (A) Šidák's multiple comparison test, (B) Tukey's multiple comparison test, or (D) Dunnett's multiple comparison test. (ns is non-significant; *<0.05, **<0.01, ***<0.001, and ****<0.0001).
