# Supplementary material for: Zinc-alpha-2-glycoprotein Secreted by Triple-Negative Breast Cancer Promotes Peritumoral Fibrosis
Source: Cancer Res Commun. 2024 Jul 5;4(7):1655–66. doi: 10.1158/2767-9764.CRC-24-0218 (PMC11224648; doi:10.1158/2767-9764.CRC-24-0218)
Supplement: Figure S4 — Supplemental Figure and Figure Legend 4 [file crc-24-0218_figure_s4_suppsf4.pdf]

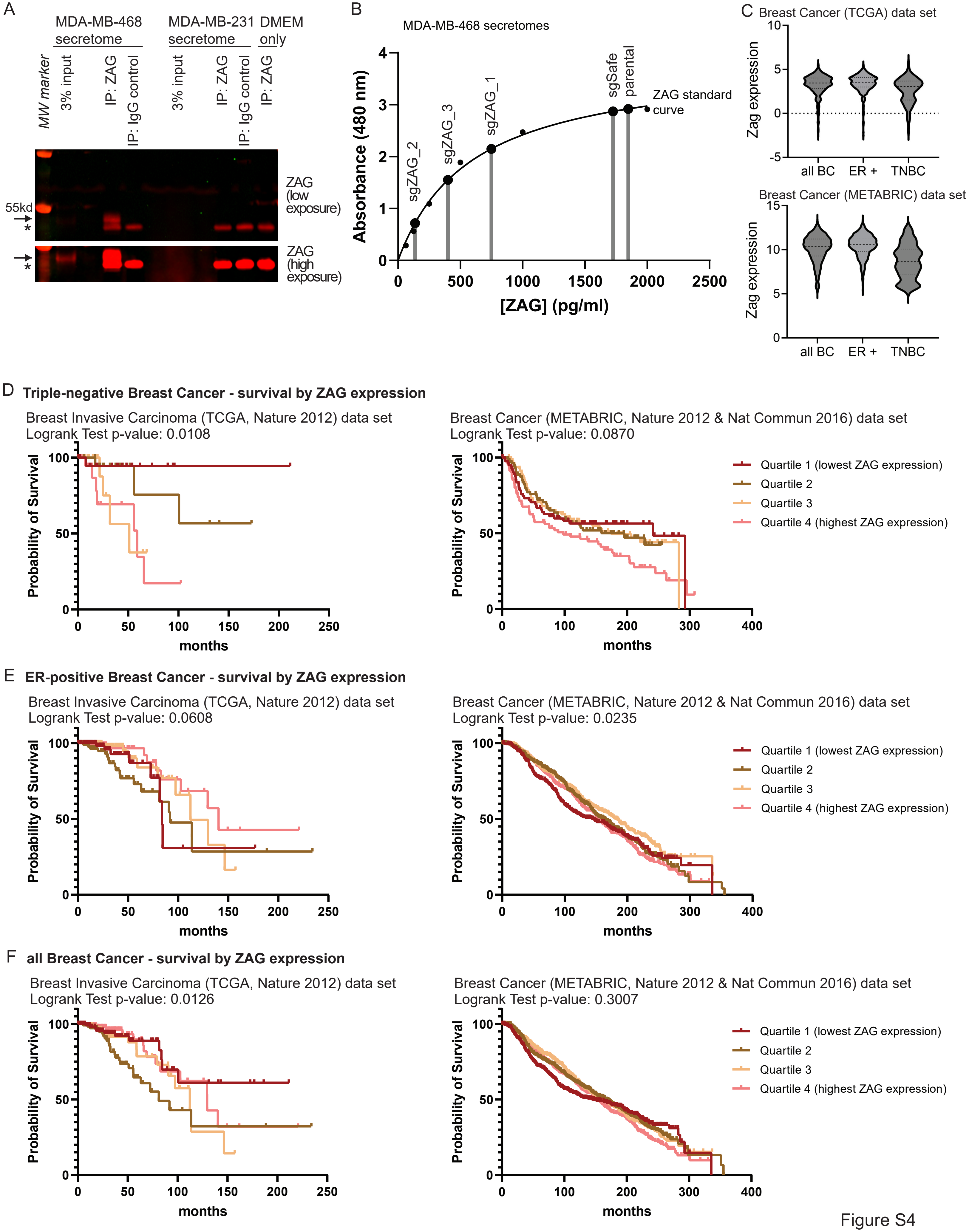
A
MDA-MB-468 secretome
MDA-MB-231 secretome
DMEM only
MW marker
3% input
IP: ZAG
IP: IgG control
3% input
IP: ZAG
IP: IgG control
IP: ZAG
55kd
*
*
ZAG (low exposure)
ZAG (high exposure)
B
MDA-MB-468 secretomes
Absorbance (480 nm)
[ZAG] (pg/ml)
sgZAG_2
sgZAG_3
sgZAG_1
sgSafe
parental
ZAG standard curve
C
Breast Cancer (TCGA) data set
Zag expression
all BC
ER +
TNBC
Breast Cancer (METABRIC) data set
Zag expression
all BC
ER +
TNBC
D
Triple-negative Breast Cancer - survival by ZAG expression
Breast Invasive Carcinoma (TCGA, Nature 2012) data set
Logrank Test p-value: 0.0108
Breast Cancer (METABRIC, Nature 2012 & Nat Commun 2016) data set
Logrank Test p-value: 0.0870
Probability of Survival
months
Quartile 1 (lowest ZAG expression)
Quartile 2
Quartile 3
Quartile 4 (highest ZAG expression)
E
ER-positive Breast Cancer - survival by ZAG expression
Breast Invasive Carcinoma (TCGA, Nature 2012) data set
Logrank Test p-value: 0.0608
Breast Cancer (METABRIC, Nature 2012 & Nat Commun 2016) data set
Logrank Test p-value: 0.0235
Probability of Survival
months
Quartile 1 (lowest ZAG expression)
Quartile 2
Quartile 3
Quartile 4 (highest ZAG expression)
F
all Breast Cancer - survival by ZAG expression
Breast Invasive Carcinoma (TCGA, Nature 2012) data set
Logrank Test p-value: 0.0126
Breast Cancer (METABRIC, Nature 2012 & Nat Commun 2016) data set
Logrank Test p-value: 0.3007
Probability of Survival
months
Quartile 1 (lowest ZAG expression)
Quartile 2
Quartile 3
Quartile 4 (highest ZAG expression)

Figure S4

Figure S4: related to Figure 3. ZAG expression and linked prognosis by breast cancer subtypes.

(A) Immunoprecipitation of endogenous ZAG in the secretomes of MDA-MB-468 and MDA-MB-231 cells. Anti-adipogenic effects of secretomes on 3T3-L1 cells was validated. ZAG is only found in the immunoprecipitated and input of MDA-MB-468 secretome (arrow, lanes 4 and 2), but not in the IgG pull down control (lane 5), ZAG pull-down of the MDA-MB-231 secretome (lanes 7-10), or ZAG pull down of the DMEM only media control (lane 11). * denotes heavy chain band. (B) Quantification of ZAG abundance in MDA-MB-468 secretome by ELISA. Quantification was determined in the secretome of three distinct cell lines depleted of ZAG, MDA-MB-468 parental cells, and MDA-MB-468 sgSafe cells (control). (C) Expression of ZAG (mRNA expression z-scores relative to all samples) in all breast cancer, TNBC, or ER-positive breast cancer patients in the TCGA data set or the Metabric data set. Data analyzed using cBioPortal. Dotted lines show quartiles in violin plots. (D-F) Patient outcome stratified by ZAG expression for TCGA data set (left) or Metabric (right) separately for (D) TNBC, (E) ER-positive breast cancer, and (F) all breast cancer. Data analyzed using cBioPortal.
