# Supplementary material for: Zinc-alpha-2-glycoprotein Secreted by Triple-Negative Breast Cancer Promotes Peritumoral Fibrosis
Source: Cancer Res Commun. 2024 Jul 5;4(7):1655–66. doi: 10.1158/2767-9764.CRC-24-0218 (PMC11224648; doi:10.1158/2767-9764.CRC-24-0218)
Supplement: Figure S5 — Supplemental Figure and Figure Legend 5 [file crc-24-0218_figure_s5_suppsf5.pdf]

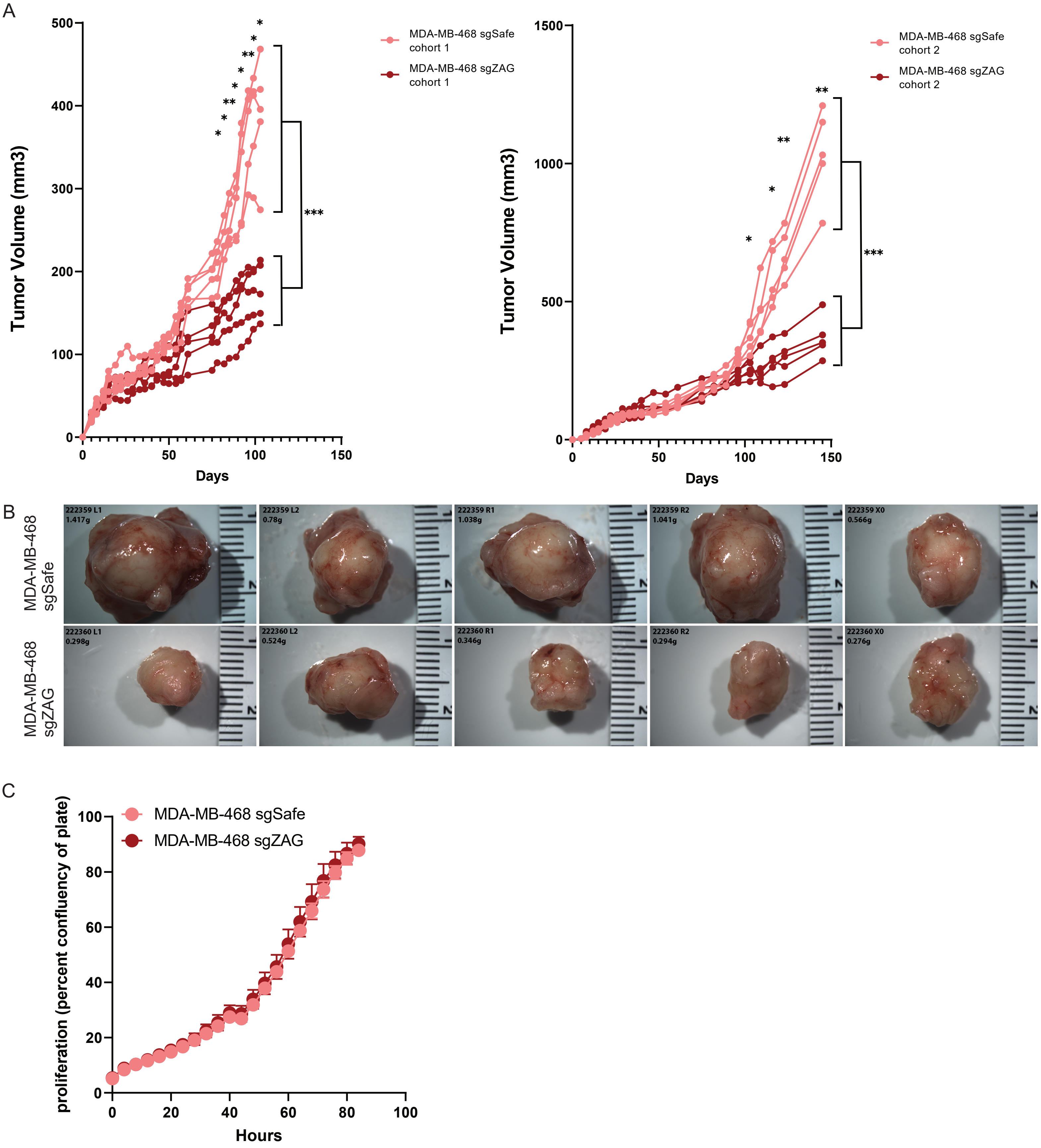

A
Tumor Volume (mm3)
Days
MDA-MB-468 sgSafe cohort 1
MDA-MB-468 sgZAG cohort 1
MDA-MB-468 sgSafe cohort 2
MDA-MB-468 sgZAG cohort 2
*
**
***
B
MDA-MB-468 sgSafe
MDA-MB-468 sgZAG
222359 L1 1.417g
222359 L2 0.78g
222359 R1 1.038g
222359 R2 1.041g
222359 X0 0.566g
222360 L1 0.298g
222360 L2 0.524g
222360 R1 0.346g
222360 R2 0.294g
222360 X0 0.276g
C
proliferation (percent confluency of plate)
Hours
MDA-MB-468 sgSafe
MDA-MB-468 sgZAG

Figure S5

Figure S5: related to Figure 4. Depletion of ZAG in MDA-MB-468 cells inhibits xenograft growth without affecting *ex vivo* proliferation.

(A) Two independent cohorts of n=5 mice. See Figure 4A for combined data of both cohorts (n=10 mice). MDA-MB-468 cells infected with sgSafe (control) or sgZAG_2 were injected into the 4th mammary fat pad of NSG female mice. One tumor per mouse. Tumor growth shown per mouse over time in cohort 1 (left) and cohort 2 (right). (B) Image of excised tumors of cohort 2. See Figure 4B for tumor weights. (C) Proliferation of MDA-MB-468 cells infected with sgSafe (control) or sgZAG_2 grown in culture (*ex vivo*). No cell-intrinsic proliferation defect was observed. (A) Lines are independent tumors/mice. P-values calculated using two-way ANOVA and followed by Šidák's multiple comparison test for individual time point. (C) Data are represented as mean ± SD (n=3 independent experiments).
