# Supplementary material for: Zinc-alpha-2-glycoprotein Secreted by Triple-Negative Breast Cancer Promotes Peritumoral Fibrosis
Source: Cancer Res Commun. 2024 Jul 5;4(7):1655–66. doi: 10.1158/2767-9764.CRC-24-0218 (PMC11224648; doi:10.1158/2767-9764.CRC-24-0218)
Supplement: Figure S6 — Supplemental Figure and Figure Legend 6 [file crc-24-0218_figure_s6_suppsf6.pdf]

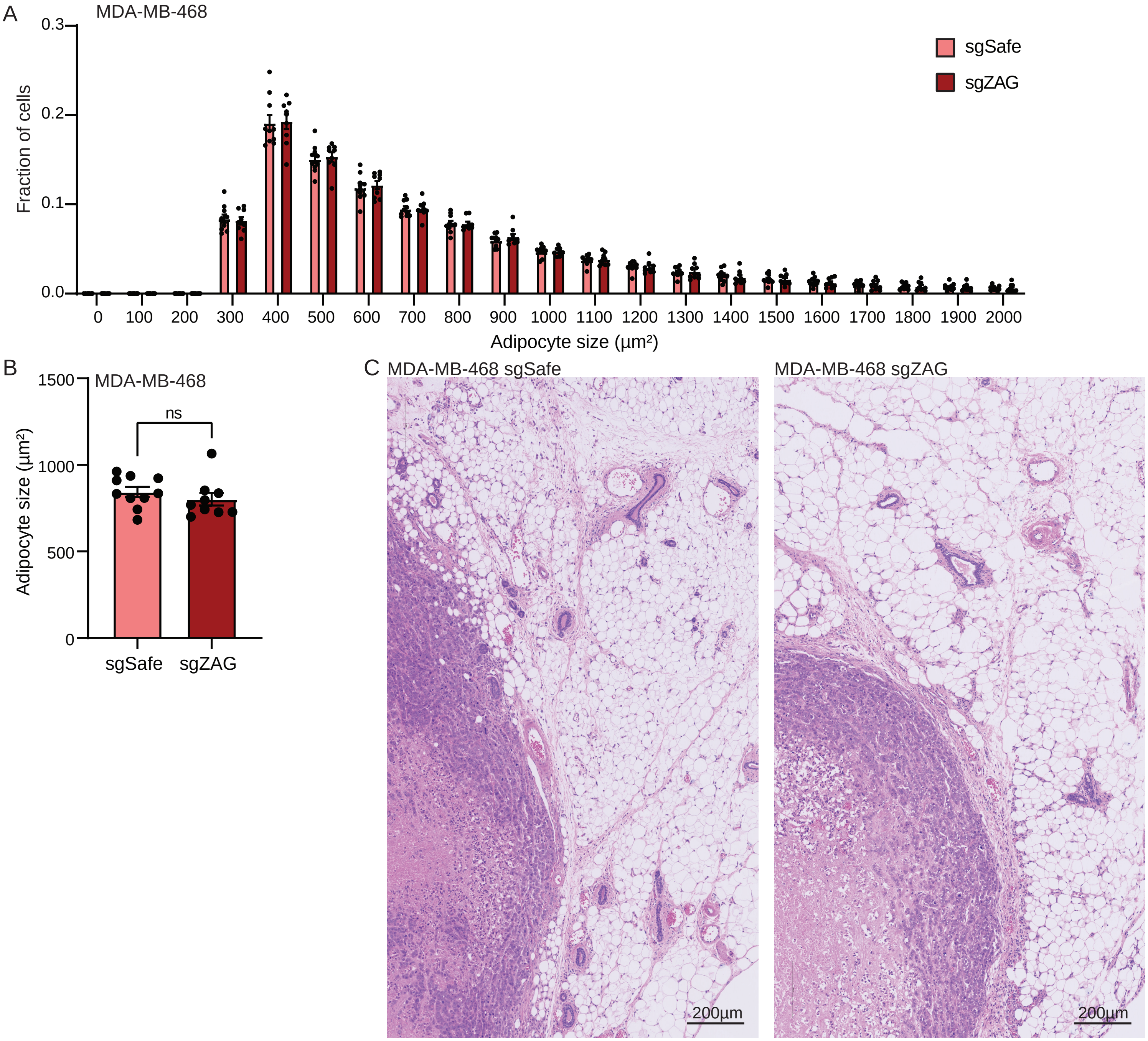
A
MDA-MB-468
sgSafe
sgZAG
Fraction of cells
0.0
0.1
0.2
0.3
0
100
200
300
400
500
600
700
800
900
1000
1100
1200
1300
1400
1500
1600
1700
1800
1900
2000
Adipocyte size (µm²)
B
MDA-MB-468
ns
Adipocyte size (µm²)
0
500
1000
1500
sgSafe
sgZAG
C
MDA-MB-468 sgSafe
MDA-MB-468 sgZAG
200µm
200µm

Figure S6

Figure S6: related to Figure 5. Depletion of ZAG in MDA-MB-468 xenografts does not affect adipocyte size in the surrounding adipose tissue.

(A) Histogram of cancer-associated adipocyte sizes. Adipocyte size was quantified in the adipose tissue surrounding MDA-MB-468 sgSafe or sgZAG_2 xenografts. Data points are binned quantification of adipocytes in sections from independent tumors/mice. (B) Average adipocyte size for all 10 tumors. The average size of cancer-associated adipocytes is not affected by ZAG depletion in MDA-MB-468 xenografts. (C) Representative H&E image of xenograft and surrounding adipose tissue. Adipocytes are lipid-filled cells. Adipocyte size was quantified for Figure S6A-B.
