# Supplementary material for: Zinc-alpha-2-glycoprotein Secreted by Triple-Negative Breast Cancer Promotes Peritumoral Fibrosis
Source: Cancer Res Commun. 2024 Jul 5;4(7):1655–66. doi: 10.1158/2767-9764.CRC-24-0218 (PMC11224648; doi:10.1158/2767-9764.CRC-24-0218)
Supplement: Table S1 — Supplemental Table 1: Primers used [file crc-24-0218_table_s1_suppst1.docx]

**Table S1: Primers used**

sgRNA oligos

| sgZAG-1F | TTG GGA CAG CCC AGT GTA GAT ATG TTT AAG AGC |
| --- | --- |
| sgZAG-1R | TTA GCT CTT AAA CAT ATC TAC ACT GGG CTG TCC CAA CAA G |
| sgZAG-2F | TTG GTC CCC GCG TTT CAG GCC CTG TTT AAG AGC |
| sgZAG-2R | TTA GCT CTT AAA CAG GGC CTG AAA CGC GGG GAC CAA CAA G |
| sgZAG-3F | TTG GGG GAC GTC TTC AAC ATG CTG TTT AAG AGC |
| sgZAG-3R | TTA GCT CTT AAA CAG CAT GTT GAA GAC GTC CCC CAA CAA G |
| sgYWHAZ-1F | TTG GTC AAC CAT GCA GAA AAC TCG TTT AAG AGC |
| sgYWHAZ-1R | TTA GCT CTT AAA CGA GTT TTC TGC ATG GTT GAC CAA CAA G |
| sgYWHAZ-2F | TTG GTG ACT GAT CGA CAA TCC CGT TTA AGA GC |
| sgYWHAZ-2R | TTA GCT CTT AAA CGG GAT TGT CGA TCA GTC ACC AAC AAG |
| sgYWHAZ-3F | TTG GAA TTC AAT GCA GGA AGG TTT AAG AGC |
| sgYWHAZ-3R | TTA GCT CTT AAA CCT TCC TGC ATT GAA TTC CAA CAA G |
| sgAHSG-1F | TTG GAC AAA GGA GCA GGA CGA GTT TAA GAG C |
| sgAHSG-1R | TTA GCT CTT AAA CTC GTC CTG CTC CTT TGT CCA ACA AG |
| sgAHSG-2F | TTG GGT GTG TTT GTA TCC CCA GTT TAA GAG C |
| sgAHSG-2R | TTA GCT CTT AAA CTG GGG ATA CAA ACA CAC CCA ACA AG |
| sgAHSG-3F | TTG GCG ATG ATC CAG AAA CTG GTT TAA GAG C |
| sgAHSG-3R | TTA GCT CTT AAA CCA GTT TCT GGA TCA TCG CCA ACA AG |
| sgCD44-1F | TTG GCT ACA GCA TCT CTC GGA GTT TAA GAG C |
| sgCD44-1R | TTA GCT CTT AAA CTC CGA GAG ATG CTG TAG CCA ACA AG |
| sgCD44-2F | TTG GGA GAA AGC TCT GAG CAT GTT TAA GAG C |
| sgCD44-2R | TTA GCT CTT AAA CAT GCT CAG AGC TTT CTC CCA ACA AG |
| sgCD44-3F | TTG GGG CAC GTG GTG ATT CCC GTT TAA GAG C |
| sgCD44-3R | TTA GCT CTT AAA CGG GAA TCA CCA CGT GCC CCA ACA AG |

| Safe (pGH119) | GTCAGTTCCTATGTGGCA |
| --- | --- |

TIDE Primers

| ZAG FW1 | TTCGAGGTGGCTGAGTAATG | TIDE PCR |
| --- | --- | --- |
| ZAG Rev1 | ATGAGGATGGGGCAATTGAT | TIDE PCR |
| ZAG Seq FW1 | CTTGAGTGAGCCTCCAGTGGT | Tide PCR sequencing |
| ZAG Seq Rev1 | TGTGGTCTGTTATTCACTGACCGT | Tide PCR sequencing |
| ZAG Seq Rev2 | GTCGTTGTAATACTCCACGATGT | Tide PCR sequencing |
| ZAG SeqFW2 | GAAAAGGCCAGTGGCAGGTCT | Tide PCR sequencing |
