# Supplementary material for: Zinc-alpha-2-glycoprotein Secreted by Triple-Negative Breast Cancer Promotes Peritumoral Fibrosis
Source: Cancer Res Commun. 2024 Jul 5;4(7):1655–66. doi: 10.1158/2767-9764.CRC-24-0218 (PMC11224648; doi:10.1158/2767-9764.CRC-24-0218)
Supplement: Supplementary Methods — Additional Materials and Methods [file crc-24-0218_supplementary_methods_suppsm.pdf]

Supplemental Materials and Methods

Cell line models

All breast cancer cell lines were purchased from ATCC in January 2021, and cultured in DMEM containing 10% FBS, 1% Pen/Strep, and 1% GlutaMAX. Cells were expanded for <4 passages to generate multiple freezings. For each experiment, cells from initial freezings were expanded and cultured for <10 passages for experiments. To obtain conditioned media, cells were grown to confluency, at which point the growth media was replaced with DMEM only to collect conditioned media. Mycoplasma testing was not routinely performed.

3T3-L1 cells were cultured in DMEM medium containing 10% Bovine Calf Serum, 1% Pen/Strep, and 1% GlutaMAX and switched to DMEM containing 10% FBS, 1% Pen/Strep, and 1% GlutaMAX during adipogenesis. 3T3-L1 cells were a gift from Prof. Peter Jackson (Stanford University) in September 2020. The ability of 3T3-L1 cells to differentiate is routinely confirmed (see in vitro adipogenesis assay). 3T3-L1 cells are cultured for <10 passages for experiments.

Murine primary preadipocytes were isolated from inguinal white adipose tissue from C57Bl/6J male mice. Primary preadipocytes were maintained and differentiated in DMEM medium containing 10% FBS, 1% Pen/Strep, and 1% GlutaMAX.

Plasmids

pMCB306 (lentiviral vector, loxP-mU6-sgRNAs-puro resistance-EGFP-loxP; RRID:Addgene_89360) and p293 Cas9-BFP were gifts from Prof. Michael Bassik (Stanford University). pCMV-VSV-G and pCMV-dR8.2 dvpr were gifts from Bob Weinberg (Addgene plasmid #8454; http://n2t.net/addgene:8454 ; RRID:Addgene_8454 and #8455; ; http://n2t.net/addgene:8455 ; RRID:Addgene_8455) (1). Lentiviral vectors containing sgRNA were generated by ligating annealed sgRNA oligonucleotides into pMCB306 vector digested with BstXI and BlpI restriction enzymes.

Cell Line generation

Lentiviral vectors carrying the gene of interest were co-transfected with pCMV-VSV-G and pCMV-dR8.2 dvpr into 293T cells using Fugene6 (Promega). Media was replaced after 24h and virus was harvested 48 and 72h post-transfection. Virus was filtered with a 0.45µm OVDF filter (Millipore) or freeze-thawed and subjected to centrifugation at 3000rpm for 10min. Cell lines were infected with virus in 10µg/ml polybrene (Millipore). Media was replaced after 24h and infected cells were isolated via FACS sorting after 48-72h post-infection.

MDA-MB-468 cells expressing Cas9-BFP were generated by infection of virus harvested from 293T cells transfected with p293 Cas9-BFP, pCMV-VSV-G and pCMV-dR8.2 dvpr. MDA-MB-468 Cas9-BFP cells were sorted for BFP positivity. To generate Crispr/Cas9 knockout cells, MDA-MB-468 Cas9-BFP cells were infected with lentivirus containing the sgRNA of interest. Primers listed in Table S1. Knockout efficiency was determined 10 days post-infection by TIDE analysis (2). Cells expressing a safe-targeting sgRNA were used as control (3). Primers listed in Table S1.

Oil Red O staining and quantification

Cells are fixed in 4% PFA/PBS for 10min at room temperature, followed by 3 rinses in PBS. Samples were incubated in 60% isopropanol for 5min at room temperature and then allowed to

dry completely. Samples are then incubated in freshly diluted 60% Oil Red O staining solution in water (stock is 0.5% Oil Red O (Sigma, 00625) in isopropanol) for 20min at room temperature, followed by 3 rinses in water. Samples were allowed to dry completely and imaged. To quantify, Oil Red O was extracted by incubating dried samples stained on the same day in 100% isopropanol for 5min at room temperature and absorbance was measured at 510nm.

Quantitative Real time PCR

RNA was extracted using the RNeasy Mini Kit (QIAGEN, 74104) and cDNA was synthesized using M-MLV Reverse Transcriptase (Invitrogen, 28025-013). Quantitative real time PCR was performed using TaqMan Probes (Invitrogen) and the TaqMan Gene Expression Master Mix (Applied Biosystems, 4369016) in 96-well MicroAmp Optical reaction plates (Applied Biosystems, N8010560). Probes used: PPARG Mm01184322; CEBPA Mm00514283; ADIPOQ Mm04933656; INHBA Mm00434339; TNC Mm00495662; ACTA2/aSMA Mm01546133; CTGF Mm01192933; NONO Mm00834875.

Sample preparation and immunoblot

Cells were lysed on ice in RIPA buffer (Thermo Scientific, J62524) containing protease inhibitor (Thermo Scientific, Pierce A32965) and phosphatase inhibitor (Thermo Scientific, Pierce A32957). Lysed cells were spun down and supernatant was incubated with NuPAGE™ LDS sample buffer (Invitrogen, NP0007) for 5 minutes at 95 ºC. Proteins were separated using NuPAGE™ 4 to 12%, Bis-Tris, 1.0–1.5 mm, Mini Protein Gels (Thermo Fisher Scientific, NP0321BOX) in NuPAGE™ MES SDS Running Buffer (Thermo Fisher Scientific NP0002), followed by transfer onto nitrocellulose membranes (BIO RAD, 1620115) using Towbin Buffer (2.5 mM Tris, 19.2 mM glycine, pH 8.3) containing 20% methanol. Membranes were blocked with blocking buffer (3% milk, TBST buffer (20 mM Tris, 150 mM NaCl, 0.1% Tween 20, pH 7.5)) for 30 minutes at room temperature, followed by incubation with primary antibody in 1% milk and in TBST buffer overnight (16 hour) at 4 ºC. Membrane was washed 3 times in TBST buffer for 10 minutes and incubated with secondary IRDye antibodies (1:5000 LI-COR) in blocking buffer (1% milk, TBST, 0.001% SDS) for 30 minutes at room temperature. Membranes were washed 3 times in TBST buffer and scanned on an Odyssey CLx Imaging System (LI-COR). The following antibodies were used: ZAG (Santa Cruz, sc-13585 1:200), P-Histone H3 (Cell signaling, 34655 1:1000), IRDye 680 RD (LiCor, 926-68072 1:2000).

ELISA

ELISA for ZAG was performed according to DuoSet ELISA development system (Catalog no. DY4764) instructions. Briefly wells were coated with 100 ul diluted capture antibody, sealed and incubated overnight at room temperature (RT). Next day, washing steps were performed as per instructions, and wells were blocked using supplied blocking buffer for 1 hour at RT. Washing steps were repeated, and wells were treated with reagent diluent and incubated for 2 hours. Wells were washed and detection antibody was added to wells, which were covered and incubated at RT for 2hr. Post-incubation, washes were performed, and working dilution of Streptavidin-HRP was added to wells, which were covered and incubated for 20min at RT, avoiding light exposure. Washes were performed, followed by addition of substrate solution and a 20 min incubation. Stop solution was added to terminate the reaction, followed by thorough mixing by tapping and measuring OD at 450 nm in a microplate reader (Biotek Neo2).

Sirius red staining

Slides with sections of tumors and surrounding adipose tissue were deparaffinized and hydrated in distilled water as described above. Slides were stained with Picro Sirius Red Staining Kit as per manufacturer's instructions (Abcam, ab150681). Slides were mounted with cover slides using Cytoseal XYL (Espredia, 8312-4).

Imaging

Slides were imaged on a Zeiss Axioscan Z1 and analyzed using NIS-Elements (NIKON) software. To quantify fibrosis, a threshold intensity for Sirius Red staining was set and the Sirius Red positive area was determined in the region of interest of adipose tissue (within 2mm surrounding the tumor). Percent positive Sirius Red was calculated per tumor/adipose tissue sample and averaged. To quantify proliferation, a threshold intensity was set for Ki67 immunofluorescence stain. Percent positive Ki67 positive nuclei was calculated per tumor samples and averaged. To quantify fibrosis using alpha SMA staining, a threshold intensity was set and alpha SMA positive area was determined within the adipose tissue region of interest (within 2mm of the tumor). Percent positive alpha SMA area was calculated per tumor/adipose tissue and averaged. Adipocyte size was quantified on hematoxylin and eosin (H&E)-stained slides using the Adiposoft (4) plugin on ImageJ (NIH) . Briefly, regions of interest were selected based on ROIs were chosen (minimum diameter of 20 pixels, max diameter of 200 pixels; pixel size 0.878). Frequency distribution of adipocyte diameter was determined per tumor/adipose tissue and averaged.

References

1. Stewart SA, Dykxhoorn DM, Palliser D, Mizuno H, Yu EY, An DS*, et al.* Lentivirus-delivered stable gene silencing by RNAi in primary cells. RNA **2003**;9:493-501
2. Brinkman EK, Chen T, Amendola M, van Steensel B. Easy quantitative assessment of genome editing by sequence trace decomposition. Nucleic Acids Res **2014**;42:e168
3. Morgens DW, Wainberg M, Boyle EA, Ursu O, Araya CL, Tsui CK*, et al.* Genome-scale measurement of off-target activity using Cas9 toxicity in high-throughput screens. Nat Commun **2017**;8:15178
4. Galarraga M, Campion J, Munoz-Barrutia A, Boque N, Moreno H, Martinez JA*, et al.* Adiposoft: automated software for the analysis of white adipose tissue cellularity in histological sections. J Lipid Res **2012**;53:2791-6
